# A Similarity Analysis of Audio Signal to Develop a Human Activity Recognition Using Similarity Networks

**DOI:** 10.3390/s17112688

**Published:** 2017-11-21

**Authors:** Alejandra García-Hernández, Carlos E. Galván-Tejada, Jorge I. Galván-Tejada, José M. Celaya-Padilla, Hamurabi Gamboa-Rosales, Perla Velasco-Elizondo, Rogelio Cárdenas-Vargas

**Affiliations:** 1 Academic Unit of Electrical Engineering, Autonomous University of Zacatecas, Jardín Juarez 147, Centro, Zacatecas 98000, Zacatecas, Mexico; alegarcia@uaz.edu.mx (A.G.-H.); gatejo@uaz.edu.mx (J.I.G.-T.); hamurabigr@uaz.edu.mx (H.G.-R.); pvelasco@uaz.edu.mx (P.V.-E.); 2 CONACyT—Academic Unit of Electrical Engineering, Autonomous University of Zacatecas , Jardín Juarez 147, Centro, Zacatecas 98000, Zacatecas, Mexico; jose.celaya@uaz.edu.mx; 3 Chemical Engineering Program, Autonomous University of Zacatecas, Ciudad Universitaria Siglo XXI, Carretera Zacatecas-Guadalajara Km. 6, Ejido La Escondida, Zacatecas 98160, Zacatecas, Mexico; rcardenasv@uaz.edu.mx

**Keywords:** human activity recognition, similarity networks, mel frequency cepstral coefficients

## Abstract

Human Activity Recognition (HAR) is one of the main subjects of study in the areas of computer vision and machine learning due to the great benefits that can be achieved. Examples of the study areas are: health prevention, security and surveillance, automotive research, and many others. The proposed approaches are carried out using machine learning techniques and present good results. However, it is difficult to observe how the descriptors of human activities are grouped. In order to obtain a better understanding of the the behavior of descriptors, it is important to improve the abilities to recognize the human activities. This paper proposes a novel approach for the HAR based on acoustic data and similarity networks. In this approach, we were able to characterize the sound of the activities and identify those activities looking for similarity in the sound pattern. We evaluated the similarity of the sounds considering mainly two features: the sound location and the materials that were used. As a result, the materials are a good reference classifying the human activities compared with the location.

## 1. Introduction

Human Activity Recognition (HAR) has been an area of great interest for the academy and the industry. This is due to the various applications that can be developed with this context information. For example, systems can be designed that support fragile persons, such as elderly or blind people to carry out their activities of daily living at home. In addition, systems based on computer vision that detect suspicious behavior of a subject in crowds of people can help detect terrorist attacks.

In literature, several technological approaches have been proposed to recognize human activities [1,2,3,4]. These activities can be divided into two types: simple activities (i.e., walking, running, climbing stairs, moving arms) and complex (or long-term) activities (which include several simple activities—for example, cooking—that could be comprised of walking and moving one’s arms [5,6]). Most of the approaches that have been proposed are characterized by sensors involved that must be carried by subjects (accelerometers, microphones, gyroscopes, biosensors, plantar pressure sensors, Radio Frequency Identification (RFID) tags, among others [7,8,9,10,11]), as well as devices embedded in their environment such as camcorders [12,13,14].

Recently, interest in the study of ambient sound has taken hold within the area of activity recognition. The use of the fingerprint of each particular sound, as well as the availability of many acoustic sensors in the gadgets used every day, allow the ambient sound to be a broad and easy access source of information to determine the human activities. For instance, Zhan et al. [15] propose an algorithm that uses a Haar wavelet for audio feature extraction and a hidden Markov model (HMM) for classification. Their algorithm is able to recognize twenty different environmental sounds related to personal and social activities (e.g., walking, making a telephone call, taking a shower, brushing one’s teeth, etc.). Their results indicate that an average activity recognition accuracy is 96.9%. Stork et al. [16] propose a recognition approach called Non-Markovian Ensemble Voting, which is able to recognize twenty-two different sounds related to human activities in a bathroom and kitchen (e.g., brushing one’s teeth, using a dishwasher). Their results indicate that this approach has an accuracy of 85% to recognize the human activities. Vacher et al. [17] propose AUDITHIS, a system that performs sound and speech analysis in a health smart home.

Many of the activities recognition approaches applied so far have good performance in the recognition of human activities, and some of them are based on feature selection [18,19,20]. Feature selection is an excellent technique that automatically selects those features in the data that are most relevant for the problem. This technique seeks to reduce the number of attributes in the dataset. However, according to Zhao et al. [21], there are some complex data types such as data streams from sensor networks, genetic data or social network analysis data that feature selection algorithms cannot handle effectively. One of the main advantages of network analysis is that it allows for observing the behavior of the studied phenomena more clearly [22,23,24]. For example, feature selection has been used in the detection of variables in genetic datasets, and it has made it possible to detect important genes in diseases such as diabetes [25] or cancer [26]. However, a recent study shows that when we want to study more closely disease and genetic interaction, it is a good choice to use similarity networks and some clustering network algorithms to identify communities and organize genes within clusters that highlight biological processes [27]. According to Costanzo et al. [27], studying the problem using similarity networks allowed them to have a more organized view of the problem and a more comprehensible view of cellular function.

In the real world, we can find networks almost everywhere, people are connected through different relationships, the web is a network of interconnected web pages, the interactions between molecular structures in a body also can be represented as a network and it can be modeled using graph structures. Even in the brain, a special type of network that is activated when performing certain activities has been identified [22,28,29].

In this paper, we propose an analysis of similarity of the natural environmental audio signals related to human activities that are carried out in a home environment. In order to achieve this, it is proposed to apply several metrics of network analysis instead of qualitative or contextual descriptors as location. These analyses include probabilistic similarity networks showing nodes (activities) and ties (similarity) to identify the potential classes. This will allow us to study more closely the degree of similarity that exists between the sound of the activities over time. It will also allow us to identify if there are patterns of behavior between the sound of the activities studied. In this paper, we show that, when applying network analysis metrics, we found interesting patterns of behavior between the sound of the activities. There are activities that emit very similar sounds at certain times and their similarity is not always related to the location. Sometimes, the similarity is more related to the materials that are used to develop those activities.

This paper is organized as follows. A brief introduction of Human Activity Recognition (HAR) and the research importance was presented in Section 1. A description of audio clips and devices used are presented in Section 2. In Section 3, methodology is presented followed by similarity analysis, and experimentation setup is presented in Section 4. In Section 5, results of similarity networks analysis are presented, and, finally, conclusions and future work are presented in Section 6.

## 2. Dataset Description

**Figure 1:**
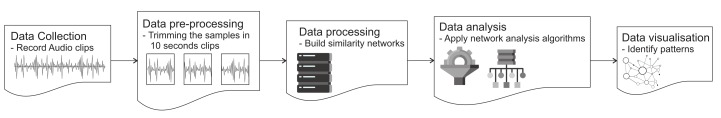
Workflow of the analysis process.

The process of analysis that we follow to carry out this research work, can be observed in Figure 1. We can see in the figure that the research consists of five main stages: data collection, data pre-processing, data processing, data analysis and data visualization. The process for carrying out each of the stages is described in the following sections.

The dataset consists of seven human activities and a collection of nonactivity sound files. These sound files are usually performed in a residential setup environment—activities such as brewing coffee, cooking, using the microwave oven, taking a shower, dish washing, hand washing, and teeth brushing. Additionally, we add the sounds not related to the activities. Table 1 shows the activities and a brief description of each. It is worth pointing out that four of these activities have a running water background sound, adding to the complexity of the HAR problem. All environmental audio recordings are available on the AmiDaMi research group page [30].

### 2.1. Recording Devices

The devices used to record the audio clips were chosen given the different specifications of the microphones embedded in each. In Table 2, the system on chip (SoC) and operating system from the selected mobile phones are shown. This information is important to explain the hardware and software characteristics involved in internal audio recording and pre-processing methods.

### 2.2. Spatial Environments

**Figure 2:**
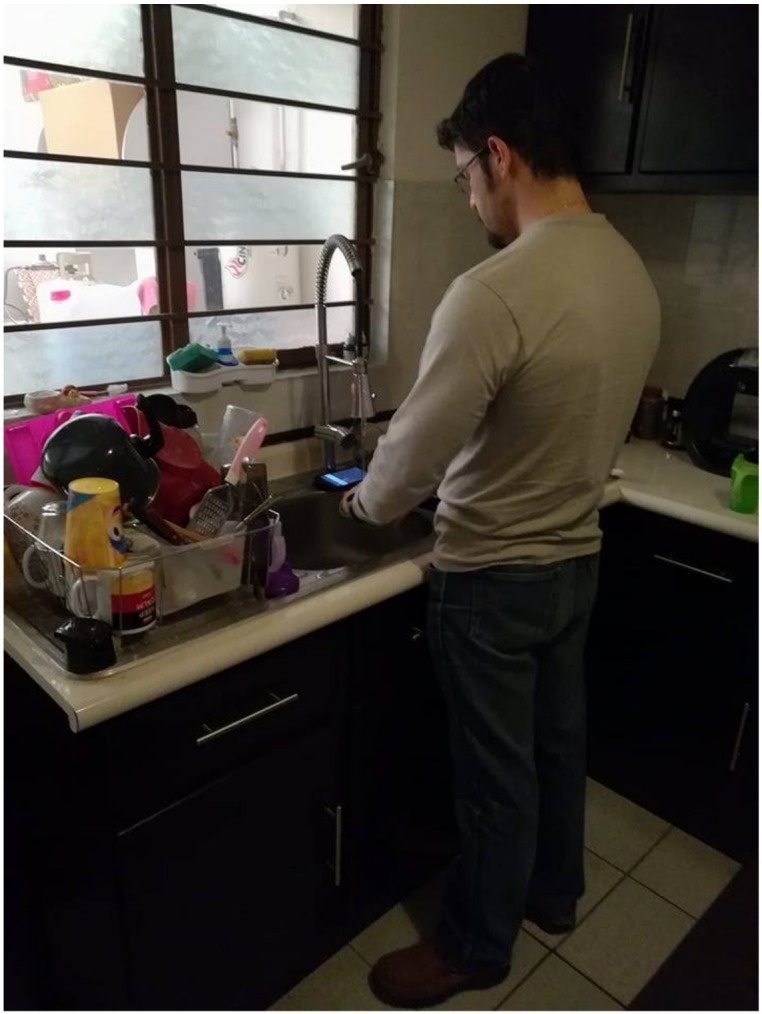
Average distance between the mobile device and activity.

With the purpose of covering a wide range of sounds, all sounds were recorded in different house locations to achieve different spatial environments (audio reflections and background sounds). Additionally, different home facilities mean different cookware, home appliances and running water reflections, and a different mobile phone that was close during the sound recording of the activity. Figure 2 shows an example of the distance that was considered between the person doing the dish-washing activity and the mobile phone that was used to record the sounds. In the image, you can see that the mobile phone is in front of the person at a short distance.

### 2.3. Meta-Data

Audio clips had a sample rate between 8000 Hz and 44,100 Hz. Mono and Stereo recordings were done depending on the device used to record the audio clip. The range of the sample rate assured that most mobile phones were able to record samples. In Table 3, the summary of meta-data for each activity performed on this dataset is shown.

### 2.4. Data Preparation

All of the audio samples have no other pre-processing other than trimming the samples in 10 s clips. No other audio processing was performed to simplify the implementation in any mobile device.

To identify acoustic descriptors from the environmental sound, Mel-Frequency Cepstral Coefficients (MFCC) are a feature widely used. We extract the MFCC from each sample. In total, 24 Cepstral coefficients per second were obtained for each sample. In Table 4, the average Mel-Frequency Cepstral Coefficients per second is shown.

## 3. Similarity Networks

In this paper, we use the audio clip recordings as our dataset. Network Analysis (NA) is applied to obtain metrics to detect and analyze similarity networks between audio records of different human activities.

There are many network analysis metrics that can be used to characterize different types of networks [31]. In this study, we analyze our similarity networks through the following network metrics: degree centrality, closeness centrality, clustering, and power law distribution. These metrics were selected because they have been widely reported in the literature. Most of the metrics of centrality (degree and closeness) have been widely used in different studies and have been found to be a key indicator to explain different social phenomena [32,33]. Specifically, the degree centrality is the simplest and most used metric.

The degree centrality of a node denotes the number of relationships that are incident with it. This metric helps to understand the influence and power of such nodes within the graph. In our case, we work with weighted graphs. This means that the links between the nodes are weighted links and the weight of the links represents the degree of similarity between the nodes. In this case, the degree centrality of a node represents the degree of similarity that the node has with respect to the other nodes present in the network.

Closeness centrality is calculated as the sum of the length of the shortest paths between one specific node and all other nodes in the network. This means that the more central a node is, the closer it is to all other nodes in the network. Clustering is another important network metric and is the most used to detect communities in the networks [34,35,36]. We applied it to detect communities between the activities in our similarity networks.

Another important phenomenon that has been identified in many complex social, biological or technological systems is that they do not follow a normal distribution. It has been found that they follow a power law distribution [37]. The power law distribution is used to describe events in which a random variable reaches high values infrequently, while low values are much more common. Some examples are the population of cities or the intensities of earthquakes. To follow a power law distribution in the case of the population of cities means, for example, that there are few cities with a large population and many cities with few population [38].

The power law distribution is very related to the probability distribution of degree over all nodes in the network, which often has a power law form compatible with the existence of high degree nodes or network hubs [39]. The objective is to identify whether, in our networks of similarity, the activities follow a power law distribution. That is, to identify if there are few activities that really have a high degree of similarity, while most have a low degree of similarity.

Through the analysis of the networks, we seek to understand more closely the behavior that exists between human activities through networks of similarity of sounds. In the similarity networks, we find that all the activities are related to each other. What makes the difference between the relations that exist between pairs of activities is defined as the degree of similarity that the activities present in the sounds that emit. When calculating the degree centrality of each of the activities, we will obtain for each second the activities that stand out the most and those that stand out less according to their similarity. When calculating the closeness centrality between activities, we will also consider the degree of similarity between them and based on those values its proximity. What is expected to be obtained in this analysis is to identify the activity or activities that are most similar in their sound to all activities analyzed.

In the analysis of clustering, we also consider the centrality degree of each activity and the similarity that exists between the activities to group them. After identifying the clusters, we will perform a more detailed analysis to observe possible clustering patterns such as location since the location is a characteristic analyzed in several HAR studies. Finally, in our last analysis of power-law distribution, the objective is to identify if there are really few activities in each analyzed second, which actually show high degrees of similarity compared to the others.

## 4. Experimentation

Our similarity networks are represented as graphs, where the nodes are the human activities described in Table 1 and the relations between the nodes represent the degree of similarity that exists between activities, taking into account the Mel-Frequency Cepstral Coefficients (MFCC) of each activity.

To measure the degree of similarity between the activities, we use the average of the values of MFCC of all activities per second shown in Table 4. To carry out a temporal analysis of the activities, we construct a similarity matrix for every second (from literature review [40,41,42,43], 10 s is reported as a recommended time that preserves information of the audio clips.). To construct each similarity matrix, we used UCINET [44] a software widely used for Social Network Analysis (SNA), for the analysis and visualization of social network data. For each second, we use the MFCC values of the activities. For example, for the first second, we use only the first column of the Table 4. This column has the Cepstral Coefficients of the eight activities during the first second (CC1). We introduce the values of the CC1 column in UCINET, and we calculate the Euclidean distance between activities’ pairs according to their CC1 values:

(1) $$ED\left(x,y\right)=\sqrt{{\left(x_{i}-y_{i}\right)}^{2}},$$

where ED means Euclidean Distance, and (*x*,*y*) refers to the activities to which their distance is being calculated. Applying the Euclidean distance between pairs of activities, we obtain the matrix of dissimilarity between the activities as shown in Table 5. We call it dissimilarity matrix because the larger the MFCC value between two activities, the more different those activities are.

The values in Table 5 is the distance that exists between pairs of activities according to their CC values in the first second. Because, for this study, we are interested in knowing the degree of similarity between activities per second, and we apply the inverse of Euclidean distance to the previous matrix:

(2) $$Similarity\left(x,y\right)=\frac{1}{ED(x,y)}.$$

The result is a similarity matrix as shown in Table 6 for the first second. We repeat the same procedure for the other 9 s, and, as a result, we obtained a similarity matrix for every second.

As we can observe, the data of the similarity matrix between the activities in Table 6 are not normalized. To normalize them, we use a scale from 0 to 100, where a value of 0 means that the activities are very different and a value of 100 means that the activities are very similar in terms of their MFCC values.

To visualize the similarity networks that are formed during each second, we use Gephi, an open-source and leading visualization software that allows analysis of the evolution of the network over time by manipulating the embedded timeline [45].

The resulting data allowed us to see the level of similarity normalized between the activities. We consider three levels of similarity in the relations between the activities, activities between 0.014286 and 0.099961 values are considered as low values of similarity, activities between 0.102028 and 0.999006 values are considered as medium values of similarity, and activities between 1.016614 and 100 values are considered as high values of similarity. For the following analyses, we consider the relationships between activities with high values of similarity.

We introduce our activities data with high values of similarity in Gephi, and we visualized it as a dynamic network with a timeline of 10 s. This allowed us to observe over time how the relations of similarity between activities were changing. The larger size of the nodes indicates the greater degree centrality of the node in terms of its similarity with other activities, the thickness of the relationships means the degree of similarity between pair of nodes: the thicker tie means greater similarity between activities and vice versa.

To analyze more closely the network of similarity, we use R, a free software environment for statistical computing and graphics [46]. In R, we visualized the network of similarity that was formed in each second. In order to observe the level of similarity between the activities per second, we performed a degree centrality analysis for each activity in each second and a closeness centrality analysis. The centrality analyses were performed with the R/igraph 0.7.1 package of the R software (R Foundation for Statistical Computing, Vienna, Austria) [47].

The degree centrality of a node *i*, D${}_{i}$ is defined as the summing up of the edge weights of the adjacent edges to the node *i*. Closeness centrality measures how many steps are required to access every other node from a given node in the network. The closeness centrality of a node is defined by the inverse of the average length of the shortest paths to/from all the other nodes in the network [47].

To observe more closely the clusters that exist between the activities according to their degree of similarity, we applied the fast greedy community algorithm that allows us to detect communities in networks. The fast greedy community tries to optimize a quality function called modularity. The modularity measures when the division in a network is a good one. Initially, in the algorithm, every node belongs to a separate community, the algorithm iterates, and stops when it is not possible to increase the modularity any more [36].

Finally, to analyze if our networks follow a power law distribution, we use the R package poweRlaw. According to Clauset et al., in a power law distribution, a variable *x* obeys a power law if it follows a probability distribution [38,48].

(3) $$p\left(x\right)=\alpha x^{-\alpha },$$

where α is a constant parameter known as the scaling parameter. The α parameter typically lies in the range 2 < α < 3, although there are some exceptions [38].

Our model was fitted using a maximum likelihood procedure and cut-off value, X${}_{min}$, it was estimated by minimizing the Kolmogorov-Smirnov (K-S) test statistic. The X${}_{min}$ is the minimum value from which the power law is satisfied.

The closer the alpha parameter to 1, the less likely it is that an activity has a similarity greater than *x*. The lower the alpha value, the greater the inequality in the degree distribution of the more similar activities.

## 5. Results

**Figure 3:**
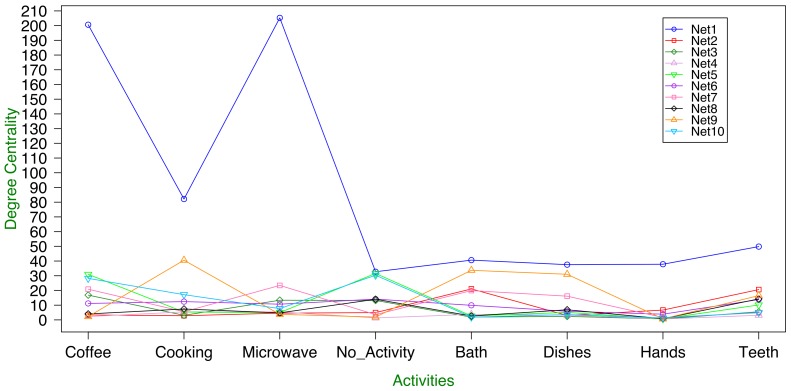
Degree centrality per second.

The results of the degree centrality analysis of each of the activities per second are shown in Figure 3. In this figure, we can observe that, in the first network (Net1), which represents the first second, the degree centrality of the activities is much higher in comparison to the other networks. The activities of brewing coffee, cooking and using the microwave oven, during this first second, have the highest degree centrality of all networks. This means that these activities in that second have a very high degree of similarity. The other activities during the first second also present a similar level of degree centrality, which makes them similar but at a lower level.

We can also observe that, in almost all networks, the degree centrality of activities is relatively low, but there are some activities that stand out from the majority in almost all the seconds, with some exceptions. In Figure 3, we can also observe the changes that exist between the degree centrality of the activities from one second to the other. For example, in the first second, there is a high degree of similarity between the activities that are performed in the kitchen (coffee, cooking, and microwave); however, in second 2, similarity is very low. This result is very important because it reflects the importance of analyzing the similarity between activities every second.

**Figure 4:**
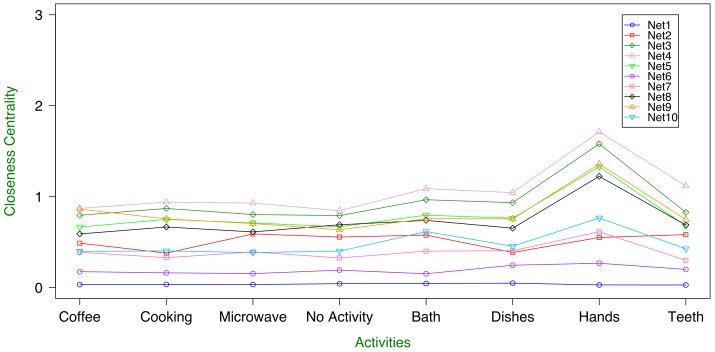
Closeness centrality per second.

In the analysis of closeness centrality that is shown in Figure 4, we can see that the activity of hand washing is the one that stands out from the others. The above means that the activity of hand washing is the activity that is closest to all other activities, according to the level of similarity in most networks. This result is particularly important because hand washing is an activity that can be performed in the kitchen or in the bathroom, and it is possible that the same sound is produced in both cases. In almost all the seconds analyzed, hand washing is closer to the other activities, which can be interpreted as an activity difficult to differentiate, since, in a way, it is very close to the other activities.

**Figure 5:**
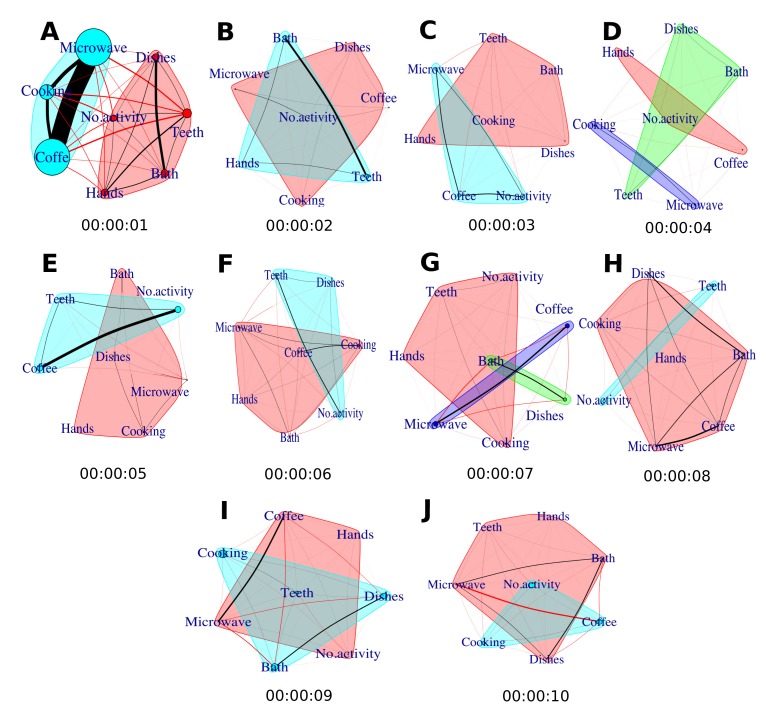
Similarity networks in 10 s., where each sub-figure represents a second.

Figure 5 allows us to observe more closely the networks of similarity between the activities per second, and the clusters that are identified according to the parameter of modularity applying the fast greedy community algorithm.

These results show that, in each second, the clusters that are forming are different. In some cases, only two large groups are formed, and, in others, we identify three groups.

By analyzing in more detail the characteristics of the activities that are grouped in each second, we could observe a particular phenomenon. The activities in some cases are grouped according to the material that is used to carry out the activity. For example, in the first second (Network A), there are two groups. The first group are activities that use a tool such as a kitchen utensil or home appliance (using the microwave, cooking and brewing coffee). The thickness of the links shows that they are activities that emit very similar sounds in this second, and, according to the location, all of these activities are done in the kitchen. The second group of the same network are all activities that only use water to wash something (dishwashing, teeth washing, taking a shower, hand washing) and are performed in different locations, in the kitchen, and in the bathroom. In second 2 (Network B), we observed almost the same phenomenon. The only difference is that the activity of dishwashing is passed to the group of activities that are done in the kitchen and that use some utensil. In Network C, the activities with the greater similarity that are grouped are those that use some utensil or apparatus and are realized in the kitchen (using the microwave and cooking). In Network D, again, the activities of cooking and using the microwave are grouped together, and, at the same time, other activities that only use water are separated into two groups. In Network D, we did not observe some pattern of similarity between the activities, perhaps because of the strong relationship between brewing coffee and no activity. Network F has grouped activities such as teeth and dishwashing, where the two use only water and are performed in different locations.

In Network G, we observed three groups, of which two of them show high similarity relations between their activities, the group where the activities of using the microwave and brewing coffee, using some utensils or apparatuses and the group taking a shower and washing dishes that use only water to wash something. In Network H, we only observed the strong relationship between using the microwave and brew coffee, and the strong relationship between taking a shower and washing dishes. In Network I, there are two groups: one highlights out the relationship between using the microwave and brewing coffee and the other highlights the relationship between taking a shower and washing dishes. In the last network, there are two groups and again we observed in one group the activities of cooking and brewing coffee, but brewing coffee had a strong relationship with using the microwave, and taking a shower and washing dishes in the other group also had a strong relationship between each other. With the above results, we could observe that, in most networks, activities are grouped according to the materials, tools, or devices that are used to carry them out.

Several studies on the recognition of human activities are based on recognizing the activities by their location. Because, in our previous results, it is observed that some activities are grouped according to the material or instruments they use to take them, we decided to measure the precision of the results by taking into consideration two criteria: we first analyze the clusters of activities according to their location, separating the activities that were performed in the kitchen (brewing coffee, cooking, using the microwave oven and dishwashing) and the activities that were performed in the bathroom (taking a shower, hand washing and brushing teeth). In a second analysis, we observed the clusters of activities according to the material that was used. We separated the activities in which only water was used to perform them (taking a shower, dish washing, hand washing and brushing teeth), and the activities that used some instrument or utensil to perform them (brewing coffee, cooking and using the microwave oven).

To measure the precision of clusters of activities according to their location, we use Equation (4):

(4) $$\mathrm{Precision}=\frac{TP}{TP+FP},$$

where *TP* means True Positive, and refers to the number of activities that were correctly clustered with other activities that were carried out in the same location, and *FP* means False Positive, and refers to the number of activities that were mistakenly clustered with other activities because they were performed at different locations. We observed the clustering of seven activities during ten seconds. No activity was not considered in this analysis. The precision for location can be seen in Equation (5):

(5) $$\mathrm{Precision}\;\mathrm{for}\;\mathrm{location}=\frac{46}{46+24}=0.65.$$

To measure the precision of clusters of activities according to the materials used, we use the same equation, where *TP* means the number of activities that were correctly clustered with other activities because they were performed only with water or because they were performed using some kitchen utensil, and *FP* means the number of activities that were mistakenly clustered with other activities because some activities in the same group used only water and some kitchen utensils. The precision for materials used can be seen in Equation (6):

(6) $$\mathrm{Precision}\;\mathrm{for}\;\mathrm{materials}\;\mathrm{used}=\frac{55}{55+17}=0.78.$$

As can be seen from the above results, the degree of precision is greater when we consider the materials used for activities such as clustering pattern, when we consider the location.

In our last analysis, our objective was to verify if our audio similarity networks followed a power law distribution. The results we obtained for each network per second are shown in Table 7. For most of the networks that follow a power law distribution, their alpha parameter lies between 2 < α < 3. In our networks, we observe that in the seconds 4, 6, 7 and 9, this parameter is not within that range. In these activities, the alpha value is very high, indicating that the inequality between the degree of similarity of the activities is really very low. The other networks show lower values of alpha, which shows that there is greater inequality in the degree of similarity of the activities. It can also be observed that, for these activities, the level of significance of the Kolmogorov-Smirnov (K-S) test is greater than 0.05, which indicates that the test accepts the hypothesis that the data of the similarity networks follow a power law distribution.

**Figure 6:**
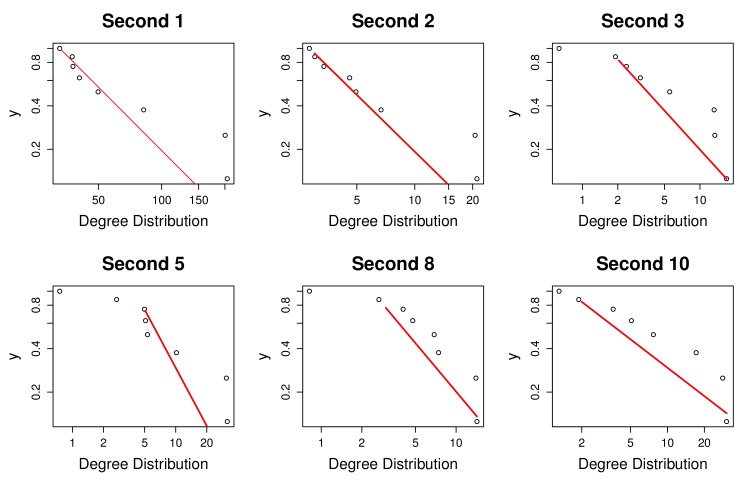
Power law distribution per second.

Figure 6 shows the plots of the degree distribution of activities per second, where the red lines are the power-law fit, starting from some X${}_{min}$ value.

The above means that, in most networks of similarity of sounds, there are only a few activities that present the greatest degree of similarity in their sound. If we observe the networks of similarity in Figure 5, we can realize that, for example, the activities that have the highest degree of similarity in several networks are microwave with coffee and dishes with bath.

## 6. Conclusions

Currently, there is a great tendency to offer or recommend customized products or services according to the preferences of the possible users. This tendency implies recognizing the preferences of the users through certain patterns of behavior. In this line, the recognition of human activities is a great challenge because, through the context, it is possible to recognize the activities carried out by users. Some studies have attempted to recognize human activities through video, motion and sound sensors, and systems designed based on these methods have not been implemented on some sites because of privacy concerns or are not very accurate [49]. For this reason, we consider that it is very important to recognize patterns in different human activities in order to identify similarities and differences between them that allow advances in the investigation of the recognition of human activities.

In this paper, we make the first approach when trying to identify patterns of behavior of human activities through acoustic data, applying network analysis metrics.

Through network analysis, we were able to identify the similarity networks that exist between different human activities, and we found interesting patterns of behavior. We observed that, at some point in time, some activities emit very similar sounds and this similarity of sound was not always related to the location where the activity was being performed, but rather to the type of materials or utensils used to perform the activity. This is a very important result because we find that location alone is not always a good reference when trying to recognize human activities. There are other aspects that should be considered such as the materials used in the activities.

Also through the analysis of networks, we were able to observe in detail the activities that presented greater similarity in their sounds over time, and we identified that, in most of the analyzed seconds, the activities follow a power law distribution.

While this method is a useful advancement in the field of recognition of human activities, further research may enhance the approach in the future—for example, applying feature selection to strengthen our results or through the recognition of more complex human activities, trying to separate the sounds emitted by the different materials and devices that are used to try to find more precise patterns of behavior.

Finally, we are aware of mixed or simultaneous activities that could lead to a misclassification of a human activity recognition. Therefore, we propose as future work a wide study using robust machine learning techniques as convolutional neural network (CNN) or a multi-staking approach to tackle this issue.

## Figures and Tables

**Table 1:** Activities’ general description.

Activity	Description
Brew coffee	Brewing coffee in the stove using coffee pots and in coffee machines.
Cook	Cooking meat and scrambled eggs in the stove.
Use microwave oven	Using several microwave ovens to heat up water and a meal.
Take a shower	Taking a shower in different environments, in some cases water fall was interrupted at intervals.
Dish washing	Dishes were washed by hand individually or in groups of different dishes, water noise is in the background.
Hand washing	Washing hands with bar soap.
Teeth brushing	Audio clips include from opening the tap to closing it.
No activity	No activity audio clips, which are mostly noises added by the device used to record (reading in silence, resting in a coach, sleeping without snoring).

**Table 2:** Selected mobile phones’ system on chip (SoC) and operating system.

Smartphone	System on Chip (SoC)	Operating System
Lanix Ilium s600	Qualcomm Snapdragon 210 MSM8909	Android 5.1
LG G Pro Lite	MediaTek MT6577	Android 4.1.2
iPhone 4	Apple A4 APL0398	iOS 4
iPhone 3GS	Samsung S5PC100	iOS 3
HTC One M7	Qualcomm Snapdragon 600 APQ8064T	Android 4.1.2

**Table 3:** Audio clips’ meta-data per activity.

Activity	Sample Rate	Encoding Format	Channels
Brew coffee	8000 Hz–44,100 Hz	m4a, amr	Stereo, Mono
Cook	44,100 Hz	m4a	Stereo
Use microwave oven	44,100 Hz	m4a	Stereo
Take a shower	44,100 Hz	m4a, mp3	Stereo
Dish washing	44,100 Hz	m4a	Stereo
Hand washing	8000 Hz–44,100 Hz	m4a, amr	Stereo, Mono
Brushing teeth	44,100 Hz	m4a	Stereo
No activity	8000 Hz–44,100 Hz	m4a, amr	Stereo, Mono

**Table 4:** Average Mel-Frequency Cepstral Coefficients (MFCC) per second during 10 s.

Activities	CC1	CC2	CC3	CC4	CC5	CC6	CC7	CC8	CC9	CC10
Brew coffee (1)	0.316	−2.689	4.503	−4.484	5.154	−2.251	−3.408	5.202	−11.333	5.621
Cook (2)	0.277	−5.423	6.552	−6.399	7.445	−2.430	−2.367	7.166	−9.044	5.802
Use microwave (3)	0.310	−1.324	4.645	−6.107	6.267	−2.802	−3.490	5.755	−7.593	5.202
No activity (4)	0.163	−1.681	4.369	−1.669	5.194	−3.713	−0.756	7.975	−5.513	5.675
Take a shower (5)	0.673	−8.038	9.892	−9.121	9.068	−3.150	−3.661	9.492	−9.094	9.757
Dish washing (6)	0.722	−4.107	8.595	−8.198	7.863	−4.416	−3.781	6.834	−8.975	7.690
Hand washing (7)	0.510	−7.697	−5.127	6.109	−2.870	−1.157	1.893	−1.583	1.180	0.961
Brushing teeth (8)	0.407	−8.097	5.246	−9.758	5.437	−3.838	−1.621	7.878	−8.824	6.750

**Table 5:** MFCC dissimilarity matrix for second 1.

Activities	1	2	3	4	5	6	7	8
1	0.000	0.039	0.007	0.154	0.357	0.405	0.194	0.090
2	0.039	0.000	0.032	0.115	0.396	0.444	0.233	0.129
3	0.007	0.032	0.000	0.147	0.364	0.412	0.200	0.097
4	0.154	0.115	0.147	0.000	0.511	0.559	0.347	0.244
5	0.357	0.396	0.364	0.511	0.000	0.048	0.163	0.267
6	0.405	0.444	0.412	0.559	0.048	0.000	0.212	0.315
7	0.194	0.233	0.200	0.347	0.163	0.212	0.000	0.103
8	0.090	0.129	0.097	0.244	0.267	0.315	0.103	0.000

**Table 6:** MFCC similarity matrix for second 1.

Activities	1	2	3	4	5	6	7	8
1	0.00	25.61	147.00	6.50	2.80	2.47	5.16	11.09
2	25.61	0.00	31.01	8.71	2.52	2.25	4.30	7.74
3	147.00	31.01	0.00	6.80	2.75	2.43	4.99	10.31
4	6.50	8.71	6.80	0.00	1.96	1.79	2.88	4.10
5	2.80	2.52	2.75	1.96	0.00	20.71	6.12	3.75
6	2.47	2.25	2.43	1.79	20.71	0.00	4.72	3.17
7	5.16	4.30	4.99	2.88	6.12	4.72	0.00	9.66
8	11.09	7.74	10.31	4.10	3.75	3.17	9.66	0.00

**Table 7:** Power law distribution statistics for audio similarity networks per second.

Second	Alpha	X${}_{\min}$	LogLik	KS.stat	KS.p
1	2.29	32.73	−38.4290	0.1789	0.9355
2	2.16	2.83	−20.3705	0.1750	0.9454
3	1.78	1.91	−20.1863	0.2512	0.6933
4	4.23	3.16	−5.3490	0.2770	0.7463
5	2.14	4.97	−18.4297	0.2467	0.7875
6	5.62	9.90	−10.8197	0.2057	0.9282
7	11.79	19.92	−4.2951	0.2361	0.9789
8	1.97	2.67	−19.4789	0.2261	0.8077
9	7.37	30.99	−7.3468	0.2320	0.9823
10	1.57	1.89	−25.2218	0.2010	0.9027
